# HDAC2 Inhibitor Valproic Acid Increases Radiation Sensitivity of Drug-Resistant Melanoma Cells

**DOI:** 10.3390/medsci7030051

**Published:** 2019-03-22

**Authors:** Bhuvanesh Sukhlal Kalal, Vinitha Ramanath Pai, Santosh Kumar Behera, Hiriyur Mallaiah Somashekarappa

**Affiliations:** 1 Department of Biochemistry, Yenepoya Medical College, Yenepoya (Deemed to be University), Mangaluru-575018, Karnataka, India; bhuvanesh611@gmail.com; 2 Center for Systems Biology and Molecular Medicine, Yenepoya Research Centre, Yenepoya (Deemed to be University), Mangaluru-575018, Karnataka, India; beheras40@gmail.com; 3 Centre for Application of Radioisotopes and Radiation Technology, Mangalore University, Mangaluru-574199, Karnataka, India; carrtmu@gmail.com

**Keywords:** cytotoxic, drug-resistance, histone deacetylase 2, melanoma, radiosensitizer, valproic acid

## Abstract

Resistance to anticancer drugs limits the effectiveness of chemotherapy in cancers. Melanoma cell lines B16F10C and A375C (parental) and B16F10R and A375R (drug-resistant sublines) were used to test radiation sensitization potential of valproic acid (VPA), an inhibitor of Histone deacetylase2 (HDAC2) and LDN193189 (BMP inhibitor). Inhibitors of other signaling pathways were tested for cross-resistance with the resistant cell lines. Cells were pretreated with low concentrations of VPA/ LDN193189 and exposed to 2 Gy radiation for radiation sensitization experiments. Assays-3-(4,5-Dimethylthiazol-2-yl)-2,5-Diphenyltetrazolium Bromide (MTT), live/dead, clonogenic, and melanin estimation were performed to test the effects of radiation sensitization. Interactions of VPA and HDAC2 were studied in silico. Dose-dependent growth inhibition was observed with all tested drugs. Radiation sensitization of melanoma cells with low dose of VPA induced synergistic cell death, decreased clonogenicity, and decreased melanin content. In silico docking showed two stable interactions between Arg39 of HDAC2 and VPA. In conclusion, pretreatment with low doses of VPA has a potential for sensitizing melanoma cells to low doses of radiation. The binding of VPA to HDAC2 reverses the drug resistance in melanoma and induces the cell death. Sensitization effects of VPA can be used for targeting drug-resistant cancers.

## 1. Introduction

Radiation therapy is a choice of treatment for most types of cancers, with over half of all cancer patients receiving this therapy during the course of treatment [1,2]. Since melanoma is considered a radioresistant tumor, there has been controversy over the use of radiotherapy for melanoma since the 1940s [3]. Several studies demonstrated that melanoma consists of heterogeneous cells which are not completely radioresistant [4,5], while other studies argued that melanoma is radioresistant [6,7,8]. Thus, radiotherapy is rarely used as a primary treatment for melanoma, although it is currently used in adjuvant, elective and palliative treatments [9,10]. Therefore, it is very essential to modulate the responsiveness of the melanoma cells to radiation for utilizing radiotherapy as an essential primary treatment. This can be done by targeting molecules that are involved in intracellular signaling events conferring radiation resistance to melanoma cells upon exposure. To target these molecules, understanding the signaling activities, associated with radiation resistance is essential.

The development of cells that are resistant to chemotherapeutic agents is a major clinical obstacle to the successful treatment of cancer, including melanoma. New novel molecules for therapy, whether chemotherapeutic or targeted, are regularly developed to combat the development of resistance to chemotherapy [11]. Though anticancer therapies bring changes in the growth of the tumor, the effect is unfortunately only temporary in most cases, since this phase reverts back to the original growth. Such treatment failures will affect the survival of patients with advanced melanoma.

Resistance to individual chemotherapeutic agents usually occur by alterations in the targets for these drugs. However, resistance can also develop more broadly to a variety of diverse unrelated antitumor drugs with different chemical structures and different mechanisms of action [12,13]. The existence of this cross-resistance suggests that many chemotherapeutic agents may or may not share the common mechanisms of resistance. Although drug resistance can be overcome using epigenetic therapies in experimental models, clinical studies of epigenetic therapies highlighted challenges for different cancers, and there is a need to identify more targeted approaches [14].

Radiation therapy is one of the most commonly used interventions in cancer therapy. However, innate or acquired radio resistance in several cancers and toxicity to normal tissues are still serious concerns. In order to enhance radiation response in cancer therapy, multiple radiosensitizers were developed and investigated for clinical use. Radiosensitizers are drugs that make cancer cells more vulnerable to radiation therapy. To date, there are no clinically relevant, effective radiosensitizers available.

Bone morphogenetic proteins (BMPs) are members of the transforming growth factor-*β* (TGF-*β*) family that involves in regulation of cell proliferation, migration, apoptosis, and differentiation. Studies on cancers indicated that malfunctions of BMP signaling pathways were more or less responsible for alteration in therapeutic response and make cancer cell non responsive to inhibitor or radiation [15]. Some of the reports show that inhibition of BMP pathways sensitize cancer cells to radiation [16,17].

Histone deacetylases (HDACs) are involved in epigenetic gene regulation through alterations of the chromatin status of DNA. Aberrant expression, dysregulation of their enzymatic activity, or imbalances between HDACs and histone acetyltransferases (HATs) will likely be engaged with the improvement and movement of malignant growth. Pharmacologic inhibition of HDACs by histone deacetylase inhibitors (HDACis) show potent antitumor activity in a panel of malignancies including melanoma. In spite of the fact that HDACis show promise as single agents, another potential for HDACis may lie in their ability to modulate the activity of other therapeutic agents. In cancers that respond poorly to chemotherapy, treatment with HDACis can increase the sensitivity of the cancer cells to other drugs and treatments such as radiotherapy. Inhibitors of HDAC such as asvorinostat, depsipeptide, MS-275, valproic acid (VPA), and trichostatin A (TSA), show additive or synergistic anticancer activity when used along with conventional chemotherapeutic drugs [18,19,20]. Valproic acid selectively modulates the activity of histone deacetylase 2 (HDAC2) and induces proteasomal degradation [21].

Several studies using HDACis show an increase in radiation and chemo sensitization effects on drug-resistant cancer cell lines [22,23]. In the present study, we investigated the antitumor activity of VPA (an HDACi) on drug-resistant melanoma cell lines. Further, the ability of HDACis to sensitize the melanoma cells to radiation was also investigated. Earlier studies reported that HDACi enhances the efficacy of radiation-induced apoptosis [19,24,25]. Therefore, we expected that pretreatment with valproic acid (VPA)would sensitize the cells and increase the cell-killing effect of radiation, irrespective of their resistance status. In addition, inhibitors of other pathways were tested for cross-resistance with the drug-resistant melanoma sublines and the most sensitive was tested along with VPA for its radiation sensitization effects.

## 2. Materials and Methods

### 2.1. Cells and Cell Culture

Murine B16F10 melanoma cells and human A375 melanoma cells were purchased from the National Centre for Cell Science (NCCS Pune, Pune, India). The cells were cultured in Dulbecco’s Modified Eagle’s Medium (Himedia, India) supplemented with 10% fetal bovine serum and antibiotics (100 U/mL penicillin G and 100 mg/mL streptomycin), in a humidified 5% CO_2_ incubator at 37 °C. Dual drug-resistant melanoma models were developed by horizontal co-targeting two pathways (mitogen-activated protein kinases/extracellular signal-regulated kinases (MEK/ERK) andphosphatidylinositol-3-kinase/mammalian target of rapamycin complex (PI3K/mTOR) pathways) with inhibitors U0126 (ERK1/2 inhibitor) and LY294002 (AKT inhibitors). The guidelines given by McDermott et al., (2014) were followed for the generation of drug-resistant model and calculation of the resistance fold factor [26]. In all experiments, control/parental cells are referred to as B16F10C and A375C, and the dual drug-resistant cells are referred to as B16F10R and A375R.

### 2.2. Chemicals, Radiation, and Treatment

LDN193189 (bone morphogenetic proteins (BMP) inhibitor; Sigma Aldrich, St. Louis, MO, USA; Cat no: SML0559), SP600125 (c-Jun N-terminal kinases (JNK) inhibitor; Calbiochem, San Diego, CA, USA; Cat no: 129566), IWP-2 (wingless/integrated (WNT) inhibitor; Calbiochem; Cat no: 662005) were dissolved in dimethyl sulfoxide (Himedia, Mumbai, India), aliquoted for single use and stored at −20 °C. VPA (HDAC inhibitor; Sigma Aldrich; Cat no: 676380) was dissolved in Dulbecco’s Modified Eagle’s Medium (DMEM, Himedia, Mumbai, India) medium immediately before use.

### 2.3. MTT Assay

Cell viability was assessed to study the of IC_50_ values of the drugs used, optimization of the radiation dose and effects radiation sensitization by 3-(4,5-dimethylthiazol-2-yl)-2,5-diphenyltetrazolium bromide (MTT) assay [27]. This assay measures the number of living cells present after MTT exposure.

In triplicates, cells were seeded into 96-well plates at 3 × 10^3^ cells per well (100 µL) for 24 h. After 24 h, fresh medium (100 µL) was added containing different concentrations of drugs or vehicle (DMSO). After incubation for 72 h, MTT solution (100 µL) was added to each well, at a final concentration of 1 mg/mL, and incubated at 37 °C for 4 h. After incubation, the media/MTT solution was aspirated and formazan crystals were dissolved in DMSO (100 μL). The absorbance was measured at 490 nm using a multi-mode microplate reader (FLUOstar Omega; BMG Labtech, Ortenberg, Germany). The 50% inhibition concentrations (IC_50_) were derived from the dose-response curve plotted with concentration of the drug (X-axis) vs. cell viability percent (Y-axis).

### 2.4. Cross-Resistance of the Drug-Resistant Cells to Inhibitors of Other Signaling Pathways and Valpronic Acid

Cells in the exponential growth phase were seeded at a density of 5×10^4^ cells per 25 cm^2^ culture flask and incubated for 24 h to allow for cell attachment and recovery. Media was aspirated and replaced with media containing the desired concentration of the drug (LDN193189/SP600125/IWP-2/VPA). MTT assay was performed after 72 h. The dose-response curves were plotted and the IC_50_ values of the drugs for the parental and resistant cells were calculated [18,28]. Inhibitory concentration that lead to a reduction in viability by 25% (IC_25_) concentrations of VPA and LDN193189 were used for the radio sensitization experiments.

### 2.5. Optimization of the Radiation Dose for Radiation Sensitization Experiment

The flasks containing A375C and B16F10C cells were irradiated with varying doses of radiation, i.e., 2, 4, 6, and 8 Gy, with gamma radiation (GC-5000, BRIT Mumbai, Mumbai, India) at the Centre for Application of Radioisotopes and Radiation Technology (CARRT), Mangalore University, India at a dose rate of 8.81 Gy/min at room temperature. To mimic the radiation treatment procedure, a batch of cultured cells were carried to the radiation room in a similar way to radiation treatment groups but were not exposed to radiation. After radiation treatment, the cells were seeded in triplicates into 96-well plate at 8 × 10^3^ cells/well (100 µL) for 24 h. MTT assay was performed for calculation of cell viability percent. The radiation dose showing 25–30% cell death was considered as optimum for the radiation sensitization experiment.

### 2.6. Sensitization of Cells with Valpronic Acid and LDN193189

The parental and drug-resistant cells of A375 and B16F10 cell lines were pretreated with an IC_25_ concentration of VPA (0.75–1.33 mM) and LDN193189 (0.78–1.5 μM) for 24 h and irradiated with 2.0 Gy radiation. The clinically safe dose of VPA is about 1–1.5 mM for radiation sensitization. Different doses have been used. For instance, Van Nifterik et al. (2012) used 0–2.5–5–7.5 mM, while Yarmohamadi et al. (2018) used 0.5–64 mM for sensitizing cancer cells to radiation [29,30].

The following experiments were done 24 h after exposure to radiation: Cytotoxicity by MTT assay, cell viability by trypan blue, live/dead assay for apoptosis, clonogenic assay for cell proliferation and melanin production.

#### 2.6.1. MTT Assay

MTT assay was performed as described earlier and the cell viability percentage was measured.

#### 2.6.2. Cell Viability by Trypan Blue Assay

The trypan blue assay is used to determine the number of viable cells present in a cell suspension [31]. The live cells possess intact cell membranes that exclude trypan blue dye and show clear cytoplasm, whereas nonviable cells will take up the dye and stain cytoplasm blue. After 24 h of radiation treatment, cells were trypsinized and counted using trypan blue (0.4% solution) under an inverted microscope (Carl Zeiss MicroImaging GmbH, Germany).

#### 2.6.3. Live/Dead Staining

Post-radiation, the drug pretreated cells were seeded in 24 well plate, 5 × 10^4^ cells/well, and incubated overnight for attachment. Cells were treated with indicated drugs for 24 h. Cells were treated with IC_25_ of VPA and LDN193189 for 24 h. Following incubation, old media was replaced with media (0.5 mL) containing dyes acridine orange (0.01 mg/mL; AO) and propidium iodide (0.01 mg/mL; PI) and incubated for 30 min at 37 °C in the dark. Propidium iodide stains dead cells red and AO stains live cells green. Cells were visualized in phase-contrast microscopy using ZOE Fluorescent Cell Imager (Bio-Rad, Hercules, CA, USA).The difference between the stained cells was assessed quantitatively [32].

#### 2.6.4. Clonogenic Survival Assay

Since VPA was effective, this assay was done with VPA treated cells. Post-radiation, the VPA pretreated cells were trypsinized and plated onto new 35 mm culture dishes at a density of 300 cells/dish and kept without the drug for an additional 12–15 days. Media was replaced every 3–4 days with fresh DMEM. Dishes were washed with 1 × phosphate buffer saline (PBS, Himedia, Mumbai, India), fixed with *methanol:acetone* (1:1 *v*/*v*) and stained with Giemsa-stain (Sigma-Aldrich) for 1 h. The remaining staining solution was removed, and dishes were washed with distilled water and dried at room temperature. Colonies (>100 cells) were counted under a microscope [33].

#### 2.6.5. Melanin Determination

For measurement of melanin content, control and drug-resistant cells (1 × 10^6^) were seeded in 30 mm tissue culture dishes and treated with IC_25_ concentrations of VPA and LDN198183 for 24 h followed by exposure to 2 Gy of gamma radiation. After incubation for a further 24 h, cells were trypsinized and counted by trypan blue exclusion method. Cell suspension (1 × 10^6^ cells) was centrifuged at 1200× *g* for 10 min, the pellet was dissolved in NaOH (0.75 M) containing DMSO (10%), and incubated for 1 h at 80 °C. The absorbance was measured at 470 nm using ultraviolet-1800 ultraviolet–visible spectrophotometer (Shimadzu Scientific Instruments Inc, Kyoto, Japan). The absorbance percentage of the various treatments was compared with the untreated control cells of both parental and resistant cell lines [34].

### 2.7. In Silico Docking of Valproic Acid on HDAC2

Crystal structure of HDAC2 with PDB ID: 5IWG having a resolution of 1.66 Å was downloaded from Protein Data Bank (PDB). Chain B and C were removed from the homotrimer for simplicity purpose. Chain A was prepared for docking using WHAT IF web interface [35]. Two sets of docking were performed using Autodock tool (v4.2, autodock.scripps.edu); the first with the known inhibitor N-(4-amino-4′-fluoro[1,1′-biphenyl]-3-yl)oxane-4-carboxamide (IWX) and the second with VPA. A rigid docking was done using IWX, to the receptor to analyze the accuracy of docking parameters for prediction of the confirmation. Following this, a flexible ligand docking was done with the similar parameters to find the binding conformation of valproic acid to HDAC2. Preparation of the receptor prior to the docking involved removing the water molecules and then adding the required Kollman’s charges. A list of active site residues for the receptor was selected based on the interaction of IWX to HDAC2, generated using PDB sum [36]. A grid box with a center coordinate of 66.845, 29.712, and 1.928 and number of points in X, Y, Z dimension of 50, 60, and 62 was created using a grid module of Autodock v.4.2 [37]. Genetic Algorithm with 500 runs was set for docking after selecting other parameters as default.

## 3. Results

### 3.1. Cross-Resistance with Inhibitors of Other Pathways and Valproic Acid

MTT assay showed a concentration-dependent reduction in cell viability of the parental and drug-resistant sublines in the presence of all the drugs tested. Drug-resistant cells showed (A375R and B16F10R) cross-resistance with all tested drugs (Table 1; Figure S1). LDN193189 had least IC_50_ values compared to the other drugs (SP600125/IWP-2). Valproic acid, a known inhibitor of HDAC2, and LDN193189 were used as sensitizers for the radiation sensitization experiment.

### 3.2. Radiation Dose Optimization with the Parental Cell Lines

**Figure 1:**
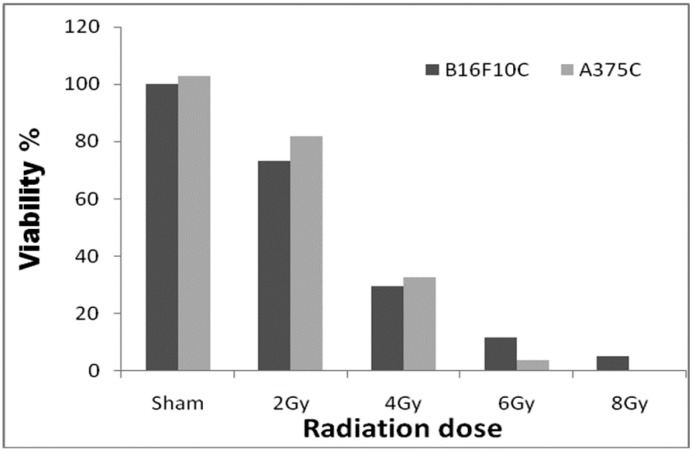
Radiation dose optimization with A375C and B16F10C cell lines. Cells were grown in T-25 flask and irradiated with 2, 4, 6, and 8 Gy gamma radiation (GC-5000, BRIT Mumbai) at the CARRT, Mangalore University, dose rate of 8.8 Gy/min at room temperature. After irradiation, cells were further incubated with drugs for 24 h. Sham served as untreated control. The dark bars represent mouse melanoma B16F10 cells and the light bars human melanoma A375 cells. The radiation dose optimization was performed only once. Therefore, it does not have an error bar.

Among the doses tested, 2.0 Gy radiation (Figure 1) showed 25–30% cell death in both A375C and B16F10C cells and was considered optimum for radiation sensitization experiments.

### 3.3. Synergistic Effect of Valproic Acid and 2Gy Radiation in Parental and Drug-Resistant Melanoma Cells

#### 3.3.1. MTT Assay

The parental and drug-resistant cells of A375 and B16F10 cells were pretreated with an IC_25_ concentration of VPA (1.33 and 0.75mM) and LDN193189(0.78 and 1.5μM) for 24 h and irradiated with 2.0 Gy radiation. The cell viability% in the A375 and B16F10, parental and drug-resistant cells with either 2.0 Gy of radiation or VPA or LDN193189, was in the range of 3–25% (Table 1; Figure S2). A375 and B16F10 cells were equally sensitive to radiation when pretreated with LDN193189 compared to VPA. The combined treatment of VPA + 2 Gy on A375C cells showed synergistic effects, i.e., cell viability% > cell viability% with 2 Gy + cell viability% with VPA, while the resistant cells showed an additive effect (cell viability% on VPA + 2Gy treatment = cell viability% with 2 Gy + cell viability% with VPA). However, the B16F10 cells pretreated with VPA followed by irradiation (2 Gy) showed a synergistic effect in both control and resistant cells. While LDN193189 pre-treated B16F10C cells showed the synergistic effect and the resistant B16F10R cells showed only additive effect (Table 2).

#### 3.3.2. Cell Viability by Trypan Blue Assay

**Figure 2:**
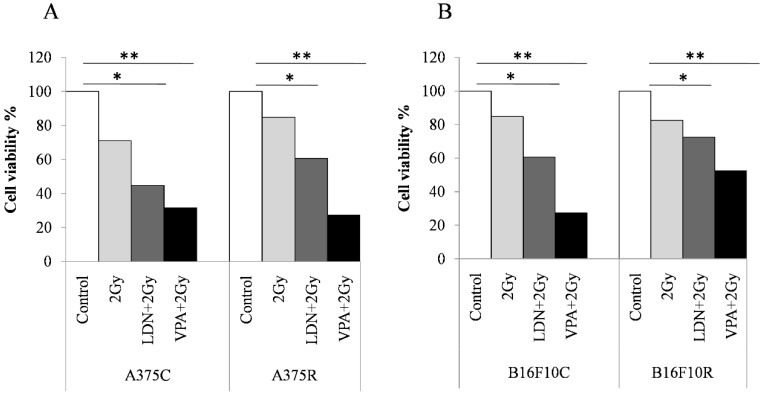
Trypan blue assay for cell viability of melanoma cells sensitized with VPA and LDN193189 for radiation treatment. Both control and drug-resistant cells were pretreated with IC_25_ dose of VPA or LDN193189. After 24 h of incubation, the flask containing cells were irradiated with gamma radiation (GC-5000, BRIT Mumbai) at the CARRT, Mangalore University, at a dose rate of 8.8 Gy/min for 14 s (equivalent to 2 Gy) at room temperature and cells were further incubated with drugs for 24 h. Following incubation, cells were trypsinized and counted by trypan blue method. Respective sham irradiation and control groups were used for comparison and data shown are mean of duplicate counts. (**A**) shows the results on human melanoma A375 and (**B**) shows the results on mouse melanoma B16F10. * *p* > 0.05, ** *p* > 0.001.

Trypan blue cell counts show (Figure 2) that there was a decrease in the cell viability% in all the treated cells when compared to the untreated cells both in the control and resistant cells of A375 and B16F10. The decrease in viable cells was more in A375R cells than in B16F10 R cells.

#### 3.3.3. Live/Dead Assay

**Figure 3:**
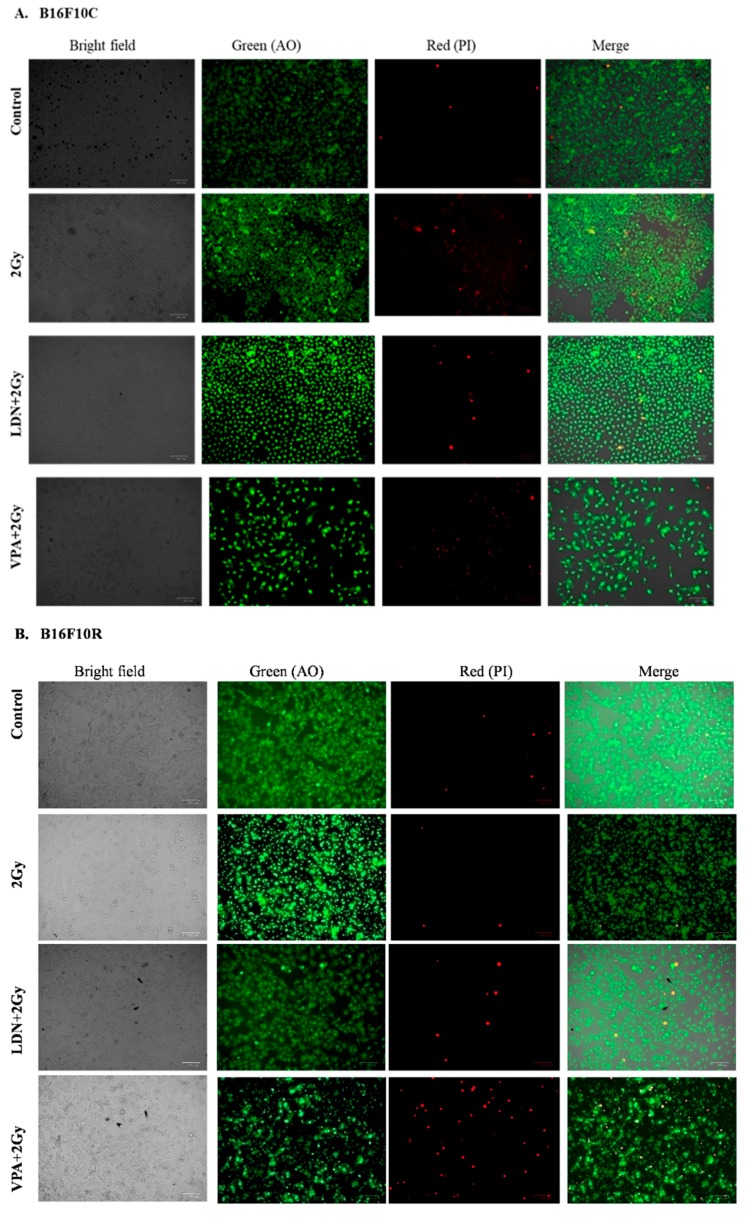
Assay of (**A**) B16F10C and (**B**) B16F10R cells with acridine orange (AO) and propidium iodide (PI) staining. Images were taken by phase-contrast microscopy using ZOE Fluorescent Cell Imager (Bio-Rad). Bright field images are from the normal light with no filter. Live cells were stained with green color (AO stain) and dead cells give red color (PI stain).

The live/dead assay showed that pretreatment of B16F10C and B16F10R with VPA followed by exposure to low dose of radiation (2 Gy) had more cell killing effect in both parental and resistant cells compared to untreated, 2 Gy treated, and LDN193189 pretreated (Figure 3).

#### 3.3.4. Clonogenic Assay

**Figure 4:**
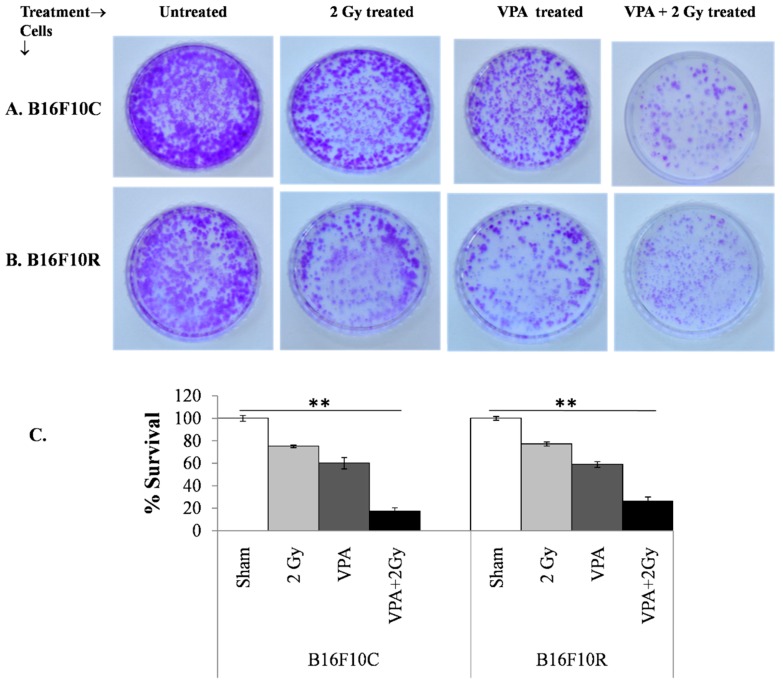
Survival assay for parental melanoma cells (B16F10C) and drug-resistant melanoma cells (B16F10R).Cells were pretreated with an IC_25_ dose of VPA (IC_25_) and incubated for 24 h. After irradiation with 2 Gy, cells were further incubated with drugs for 24 h. The cells were trypsinized and platted (300 cells/dish) and incubated for 12–15 days. The colonies in control B16F10C (**A**) and drug-resistant B16F10R (**B**) were counted and respective % survival was calculated with the sham considered as 100% and plotted in (**C**). % survival = (number of colonies in treated plate/number of colonies in untreated plate) × 100. ** *p* > 0.001.

The clonogenic survival assay also confirmed that the pretreatment of a low dose of VPA (2 mM) followed by exposure to low dose of radiation (2 Gy) increased cell death significantly (with statistical significance *p* < 0.001) in B16F10C and B16F10R cells (Figure 4A,B). The cell death was synergistic in the VPA pretreated + 2 Gy exposed cells (Figure 4C).

#### 3.3.5. Melanin Concentration

**Figure 5:**
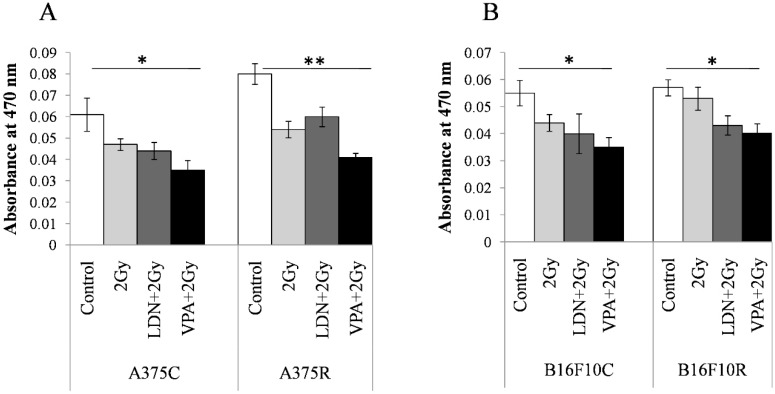
Content in the resistant cells sensitized with VPA/LDN193189 before radiation. Cells were pretreated with IC_25_ dose of VPA or LDN193189 and were irradiated. After 24 h of incubation, the flask containing cells were irradiated with gamma radiation (GC-5000, BRIT Mumbai) at the CARRT, Mangalore University, at a dose rate of 8.8 Gy/min for 14 s (equivalent to 2 Gy) at room temperature and cells were further incubated with drugs for 24 h. Following incubation, cells were lysed and the color due to melanin release was measured at 470 nm. Respective sham irradiation and control (untreated) groups were used for comparison and data shown are mean ± standard deviation (SD) of three replicate wells. (**A**) shows the results on human melanoma A375and (**B**) shows the results on mouse melanoma B16F10. * *p* > 0.05, ** *p* > 0.001.

The concentration of melanin was estimated by the differences in the absorbance at 470 nm between the parental and treated cells. The absorbance at 470 nm was maximum for all untreated cells in VPA + 2 Gy treated cells (Figure 5). The absorbance values decrease in treatment with 2 Gy, LDN + 2Gy, and VPA + 2Gy as compared to the untreated controls of all the four cell lines. Pretreatment of VPA + 2 Gy radiation showed the least absorbance indicating a maximum decrease in the concentration of melanin among all the treatments.

### 3.4. In SilicoDocking of Valproic Acid on HDAC2

**Figure 6:**
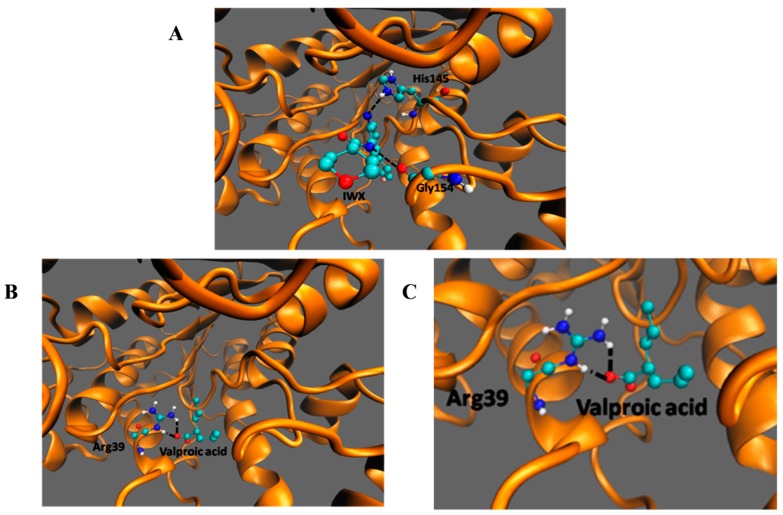
Docking of IWX, and VPA, with HDAC2.Molecular interaction of HDAC2 with (**A**) IWX inhibitor, docking control, (**B**,**C**) VPA. The black dashed lines indicate the interactions of the ligand with the labeled amino acid residues.

Among the listed interactions generated from PDBsum, two H-bonds involving His145 and Gly154 (Figure 6A) with an energy of −9.5 kcal/moL were generated between HDAC2 and its inhibitor IWX (used as a standard to check for reliability of the docking). The negative energy shows the stability of the interaction. There was no interaction between VPA and His145 and Gly146 of HDAC2 as seen in the case of the inhibitor IWX. However, valproic acid bound deeper in the enzyme and made an H-bond with Arg39 (Figure 6B,C). The interaction of HDAC2-VPA complex is stabilized by energies of −4.61 kcal/mol. This docking study shows that although the binding energy of VPA to HDAC2 is comparatively more than the interaction of IWX to HDAC2, VPA can make interactions with Arg39.

## 4. Discussion

In the present study, melanoma cells lines parental (B16F10C and A375C) and drug-resistant melanoma sublines (B16F10R and A375R) were used for testing the efficacy of VPA and LDN193189 as sensitizers for radiation treatment and VPA was more effective as a sensitizer to radiation treatment.

These drug-resistant melanoma sublines showed cross-resistance with other drugs—inhibitors of BMP, JNK, and WNT pathways. Drug-resistance remains as a significant obstacle in the achievement of efficient chemotherapy [11]. Changes in chemotherapy do not give any benefits as cancer cells, since cells that acquire resistance to one anticancer drug may also become simultaneously resistant to multiple drugs, which is referred to as multidrug-resistance or cross-resistance [38]. The findings of the present study demonstrate that the drug-resistant models of this study are cross-resistant to other drugs, i.e., inhibitors of other signaling pathways LDN193189 (BMP) > SP6001250 (JNK) > IWP-2 (WNT), and HDAC2 inhibitor (VPA), suggesting that treatment of already resistant cells with these drugs will not offer any therapeutic benefit.

Epigenetics (heritable nonstructural changes in gene expression) aberrations are one of the major mechanisms that push chemotherapy toward acquired resistance. Previous studies revealed the association of HDAC2 overexpression with metastasis, progression, and multidrug resistance [39,40,41]. Targeting these drug-resistant cells with HDACis is effective in inducing cell death, however, it requires a higher concentration drug dose that may lead to cellular toxicity and side effects [23]. Histone deacetylase inhibitors have the property of enhancing the anticancer activity of numerous chemotherapeutic agents and enhance the cytotoxic effect of radiation [18,42]. Valproic acid is a very well-established anticancer drug which inhibits both classes I and II HDACs with resultant hyperacetylation of histone H3 and H4. In cancers that respond poorly to chemotherapy, pretreatment with VPA increases the sensitivity of the cancer cells to other drugs and treatments such as chemotherapy or radiotherapy while protecting the normal cells [43]. These sensitization effects are mediated by increasing the acetylation of core histones, resulting in an open chromatin configuration that is more accessible to DNA-targeting agents, imbalance the regulation of pro- and anti-apoptotic genes, i.e., shifting the balance towards apoptosis, inducing reactive oxygen species production, and inhibiting angiogenesis [22].

The present study shows that VPA has synergistic effects in enhancing cell killing by radiation in both parental and drug-resistant cancer cells. This indicates that inhibiting the HDAC2 activity, which maybe one of the underlying mechanisms for drug-resistance in melanoma cells, is potentially helpful in controlling drug-resistant cancer. In silico docking showed VPA binds to HDAC2 by interacting with Arg39, which is known to bind to other inhibitors also [21]. Although the binding energy of VPA to HDAC2 is comparatively more than that of IWX to HDAC2, VPA can make two interactions with Arg39. Unlike the interaction of IWX, VPA interaction is deeper in the enzyme and makes an H-bond with Arg39. Arg39 is a crucial binding residue for HDAC2 and forms hydrogen bond interactions with several other inhibitors of HDAC2 [44]. The stable interaction of VPA with HDAC2 may interfere with its activity of deacetylation [45]. Therefore, the inhibition of HDAC2 by VPA reverses the sensitivity of cancer cells to chemo and radiation therapy. Valproic acid reverses the drug resistance by forming DNA-platinum adducts and downregulates the efflux transporter ATP-binding cassette subfamily C member 2 (ABCC2) and the DNA repair gene *ERCC1* [23]. It suppresses the homologous recombination repair and synchronizes cells to G1 phase, thereby increasing the radiation sensitivity [42]. This study shows that the pretreatment of low dose of VPA enhanced the radiation damage in both parental and drug-resistant cells significantly than untreated cells. Furthermore, the clonogenic survival assay also shows that VPA pretreatment reduces the colony formation compared to untreated cells. The pretreatment with a critically safe, low concentration of VPA [46] enhances the efficiency of radiation treatment probably by the accumulation of radiation (IR)-induced DNA double-strand breaks, and increase the cell radio sensitivity.

## 5. Conclusions

Melanoma is known to be radioresistant and it can quickly acquire resistance to chemotherapeutic drugs. Histone deacetylase 2 is known to play a role in drug resistance. Valproic acids is an inhibitor of HDAC2 and a known chemosensitizer. The pre-treatment of the drug-resistant cells with a low dose of VPA would sensitize the drug resistance melanoma cells and make them more susceptible to radiation-induced cell death.

## Figures and Tables

**Table 1:** Values and fold resistance of all the inhibitors used on A375 and B16F10 models.

Sl. No.	Inhibitor	Human Melanoma Cells (A375)			Mouse Melanoma Cells (B16F10)		
		IC_50_		Fold Resistance	IC_50_		Fold Resistance
		Control	Resistant		Control	Resistant	
1.	**LDN193189 (μM)**	1.5 ± 0.4	3.1 ± 0.8	2.03	2.8 ± 0.3	4.6 ± 0.5	1.65
2.	**SP600125 (μM)**	5.6 ± 1.3	8.0 ± 1.0	1.42	5.2 ± 0.9	8.3 ± 1.5	1.59
3.	**IWP-2 (μM)**	12.8 ± 1.9	12.1 ± 1.1	0.95	73.3 ± 2.5	99.1 ± 2.7	1.35
4.	**VPA (mM)**	2.59 ± 0.1	3.56 ± 0.4	1.37	1.44 ± 0.1	1.91 ± 0.1	1.32

IC_50_: concentration of drug which shows 50% growth inhibition. The fold resistance was calculated from IC_50_ values using the following formula: Fold resistance = IC_50_ drug-resistant cells/IC_50_ parental cells. The IC_50_ values were calculated from the dose-response curves in Figure S1 (for LDN193189, SP600125, IWP2, and valproic acid (VPA)).

**Table 2:** Effect of drug, radiation, and combination treatment on the B16F10 and A375 cells.

Sl. No.	Cells	Treatment	Viability (%)				
			2 Gy	LDN	VPA	2Gy + LDN	2Gy + VPA
1.	A375	Control	80	77	78	67	46 **
		Resistant	81	75	76	71	57 *
2.	B16F10	Control	92	77	76	65 **	43 **
		Resistant	97	79	80	75 *	61 **

Drugs used are LDN193189 (LDN) and valproic acid (VPA) Radiation dose was 2 Gy. Effect of the various treatments was studied in terms of cell death%. * Additive effect (sum of the effect of 2 Gy and VPA/LDN193189); ** Synergistic effect (more than the additive effect of 2Gy and VPA/LDN193189).

## Supplementary Materials

The following are available online at https://www.mdpi.com/2076-3271/7/3/51/s1, Figure S1: Cytotoxic effect of LDN193189, SP600125 IWP-2, and VPA on A375 and B16F10 cell lines. Figure S2: Radiation sensitization effects of VPA and LDN193189 on A375 and B16F10 cell lines.

## References

[1] Jaffray D.A., Gospodarowicz M.K., Gelband H., Jha P., Sankaranarayanan R., Horton S. (2015). Radiation Therapy for Cancer. Cancer: Disease Control Priorities.

[2] Owen J.B., Coia L.R., Hanks G.E. (1992). Recent patterns of growth in radiation therapy facilities in the United States: A patterns of care study report. Int. J. Radiat. Oncol. Biol. Phys..

[3] Khan M.K., Khan N., Almasan A., Macklis R. (2011). Future of radiation therapy for malignant melanoma in an era of newer, more effective biological agents. OncoTargets Ther..

[4] Somasundaram R., Villanueva J., Herlyn M. (2012). Intratumoral heterogeneity as a therapy resistance mechanism: Role of melanoma subpopulations. Adv. Pharmacol..

[5] Sambade M.J., Peters E.C., Thomas N.E., Kaufmann W.K., Kimple R.J., Shields J.M. (2011). Melanoma cells show a heterogeneous range of sensitivity to ionizing radiation and are radiosensitized by inhibition of B-RAF with PLX-4032. Radiother. Oncol..

[6] Pak B.J., Lee J., Thai B.L., Fuchs S.Y., Shaked Y., Ronai Z., Kerbel R.S., Ben-David Y. (2004). Radiation resistance of human melanoma analysed by retroviral insertional mutagenesis reveals a possible role for dopachrome tautomerase. Oncogene.

[7] Petrovic I., Ristic-Fira A., Todorovic D., Koricanac L., Valastro L., Cirrone P., Cuttone G. (2010). Response of a radioresistant human melanoma cell line along the proton spread-out Bragg peak. Int. J. Radiat. Biol..

[8] Ristic-Fira A.M., Todorovic D.V., Koricanac L.B., Petrovic I.M., Valastro L.M., Cirrone P.G., Raffaele L., Cuttone G. (2007). Response of a human melanoma cell line to low and high ionizing radiation. Ann. N. Y. Acad. Sci..

[9] Shinsato Y., Hanada T., Kisanuki T., Yonezawa H., Yunoue S., Yoshioka T., Hanaya R., Tokimura H., Hirano H., Arita K. (2012). Primary malignant melanoma in the pineal region treated without chemotherapy. Surg. Neurol. Int..

[10] Khan N., Khan M.K., Almasan A., Singh A.D., Macklis R. (2011). The evolving role of radiation therapy in the management of malignant melanoma. Int. J. Radiat. Oncol. Biol. Phys..

[11] Kalal B.S., Upadhya D., Pai V.R. (2017). Chemotherapy Resistance Mechanisms in Advanced Skin Cancer. Oncol. Rev..

[12] Hu Q., Baeg G.H. (2017). Role of epigenome in tumorigenesis and drug resistance. Food Chem. Toxicol..

[13] Rastgoo N., Abdi J., Hou J., Chang H. (2017). Role of epigenetics-microRNA axis in drug resistance of multiple myeloma. J. Hematol. Oncol..

[14] Li J., Hao D., Wang L., Wang H., Wang Y., Zhao Z., Li P., Deng C., Di L.J. (2017). Epigenetic targeting drugs potentiate chemotherapeutic effects in solid tumor therapy. Sci. Rep..

[15] Jiramongkolchai P., Owens P., Hong C.C. (2016). Emerging roles of the bone morphogenetic protein pathway in cancer: Potential therapeutic target for kinase inhibition. Biochem. Soc. Trans..

[16] Madhav A., Andres A., Duong F., Mishra R., Haldar S., Liu Z., Angara B., Gottlieb R., Zumsteg Z.S., Bhowmick N.A. (2018). Antagonizing CD105 enhances radiation sensitivity in prostate cancer. Oncogene.

[17] Hardee M.E., Marciscano A.E., Medina-Ramirez C.M., Zagzag D., Narayana A., Lonning S.M., Barcellos-Hoff M.H. (2012). Resistance of glioblastoma-initiating cells to radiation mediated by the tumor microenvironment can be abolished by inhibiting transforming growth factor-beta. Cancer Res..

[18] Kalal B.S., Pai V.R., Upadhya D. (2018). Valproic acid reduces tumor cell survival and proliferation with inhibitors of downstream molecules of epidermal growth factor receptor pathway. J. Pharmacol. Pharmacother..

[19] Saito K., Funayama T., Yokota Y., Murakami T., Kobayashi Y. (2017). Histone Deacetylase Inhibitors Sensitize Murine B16F10 Melanoma Cells to Carbon Ion Irradiation by Inducing G1 Phase Arrest. Biol. Pharm. Bull..

[20] Castro-Galache M.D., Ferragut J.A., Barbera V.M., Martin-Orozco E., Gonzalez-Ros J.M., Garcia-Morales P., Saceda M. (2003). Susceptibility of multidrug resistance tumor cells to apoptosis induction by histone deacetylase inhibitors. Int. J. Cancer.

[21] Kramer O.H., Zhu P., Ostendorff H.P., Golebiewski M., Tiefenbach J., Peters M.A., Brill B., Groner B., Bach I., Heinzel T. (2003). The histone deacetylase inhibitor valproic acid selectively induces proteasomal degradation of HDAC2. EMBO J..

[22] Yang C., Choy E., Hornicek F.J., Wood K.B., Schwab J.H., Liu X., Mankin H., Duan Z. (2011). Histone deacetylase inhibitor PCI-24781 enhances chemotherapy-induced apoptosis in multidrug-resistant sarcoma cell lines. Anticancer Res..

[23] To K.K., Tong W.S., Fu L.W. (2017). Reversal of platinum drug resistance by the histone deacetylase inhibitor belinostat. Lung Cancer.

[24] Cerna T., Hrabeta J., Eckschlager T., Frei E., Schmeiser H.H., Arlt V.M., Stiborova M. (2018). The Histone Deacetylase Inhibitor Valproic Acid Exerts a Synergistic Cytotoxicity with the DNA-Damaging Drug Ellipticine in Neuroblastoma Cells. Int. J. Mol. Sci..

[25] Zhou Y., Xu Y., Wang H., Niu J., Hou H., Jiang Y. (2014). Histone deacetylase inhibitor, valproic acid, radiosensitizes the C6 glioma cell line in vitro. Oncol. Lett..

[26] McDermott M., Eustace A.J., Busschots S., Breen L., Crown J., Clynes M., O’Donovan N., Stordal B. (2014). In vitro Development of Chemotherapy and Targeted Therapy Drug-Resistant Cancer Cell Lines: A Practical Guide with Case Studies. Front. Oncol..

[27] Mosmann T. (1983). Rapid colorimetric assay for cellular growth and survival: Application to proliferation and cytotoxicity assays. J. Immunol. Methods.

[28] Kalal B.S., Fathima F., Pai V.R., Sanjeev G., Krishna C.M., Upadhya D. (2018). Inhibition of ERK1/2 or AKT Activity Equally Enhances Radiation Sensitization in B16F10 Cells. World J. Oncol..

[29] Van Nifterik K.A., Van den Berg J., Slotman B.J., Lafleur M.V., Sminia P., Stalpers L.J. (2012). Valproic acid sensitizes human glioma cells for temozolomide and gamma-radiation. J. Neurooncol..

[30] Yarmohamadi A., Asadi J., Gharaei R., Mir M., Khoshnazar A.K. (2018). Valproic acid, a histone deacetylase inhibitor, enhances radiosensitivity in breast cancer cell line. J. Radiat. Cancer. Res..

[31] Strober W. (2001). Trypan blue exclusion test of cell viability. Curr. Protoc. Immunol..

[32] Bank H.L. (1988). Rapid assessment of islet viability with acridine orange and propidium iodide. In Vitro Cell. Dev. Biol..

[33] Rafehi H., Orlowski C., Georgiadis G.T., Ververis K., El-Osta A., Karagiannis T.C. (2011). Clonogenic assay: Adherent cells. J. Vis. Exp. JoVE.

[34] Lee J.H., Chen H., Kolev V., Aull K.H., Jung I., Wang J., Miyamoto S., Hosoi J., Mandinova A., Fisher D.E. (2014). High-throughput, high-content screening for novel pigmentation regulators using a keratinocyte/melanocyte co-culture system. Exp. Dermatol..

[35] Vriend G. (1990). WHAT IF: A molecular modeling and drug design program. J. Mol. Graph..

[36] Laskowski R.A., Chistyakov V.V., Thornton J.M. (2005). PDBsum more: New summaries and analyses of the known 3D structures of proteins and nucleic acids. Nucleic Acids Res..

[37] Morris G.M., Huey R., Lindstrom W., Sanner M.F., Belew R.K., Goodsell D.S., Olson A.J. (2009). AutoDock4 and AutoDockTools4: Automated docking with selective receptor flexibility. J. Comput. Chem..

[38] Hosokawa M., Saito M., Nakano A., Iwashita S., Ishizaka A., Ueda K., Iwakawa S. (2015). Acquired resistance to decitabine and cross-resistance to gemcitabine during the long-term treatment of human HCT116 colorectal cancer cells with decitabine. Oncol. Lett..

[39] Jung K.H., Noh J.H., Kim J.K., Eun J.W., Bae H.J., Xie H.J., Chang Y.G., Kim M.G., Park H., Lee J.Y. (2012). HDAC2 overexpression confers oncogenic potential to human lung cancer cells by deregulating expression of apoptosis and cell cycle proteins. J. Cell. Biochem..

[40] Huang W.T., Tsai Y.H., Chen S.H., Kuo C.W., Kuo Y.L., Lee K.T., Chen W.C., Wu P.C., Chuang C.Y., Cheng S.M. (2017). HDAC2 and HDAC5 Up-Regulations Modulate Survivin and miR-125a-5p Expressions and Promote Hormone Therapy Resistance in Estrogen Receptor Positive Breast Cancer Cells. Front. Pharmacol..

[41] Zhao H., Yu Z., Zhao L., He M., Ren J., Wu H., Chen Q., Yao W., Wei M. (2016). HDAC2 overexpression is a poor prognostic factor of breast cancer patients with increased multidrug resistance-associated protein expression who received anthracyclines therapy. Jpn. J. Clin. Oncol..

[42] Gerelchuluun A., Maeda J., Manabe E., Brents C.A., Sakae T., Fujimori A., Chen D.J., Tsuboi K., Kato T.A. (2018). Histone Deacetylase Inhibitor Induced Radiation Sensitization Effects on Human Cancer Cells after Photon and Hadron Radiation Exposure. Int. J. Mol. Sci..

[43] Thotala D., Karvas R.M., Engelbach J.A., Garbow J.R., Hallahan A.N., DeWees T.A., Laszlo A., Hallahan D.E. (2015). Valproic acid enhances the efficacy of radiation therapy by protecting normal hippocampal neurons and sensitizing malignant glioblastoma cells. Oncotarget.

[44] Kandakatla N., Ramakrishnan G. (2014). Ligand Based Pharmacophore Modeling and Virtual Screening Studies to Design Novel HDAC2 Inhibitors. Adv. Bioinform..

[45] Ganai S.A., Abdullah E., Rashid R., Altaf M. (2017). Combinatorial In Silico Strategy towards Identifying Potential Hotspots during Inhibition of Structurally Identical HDAC1 and HDAC2 Enzymes for Effective Chemotherapy against Neurological Disorders. Front. Mol. Neurosci..

[46] Liu G., Wang H., Zhang F., Tian Y., Tian Z., Cai Z., Lim D., Feng Z. (2017). The Effect of VPA on Increasing Radiosensitivity in Osteosarcoma Cells and Primary-Culture Cells from Chemical Carcinogen-Induced Breast Cancer in Rats. Int. J. Mol. Sci..

