# Supplementary material for: HDAC2 Inhibitor Valproic Acid Increases Radiation Sensitivity of Drug-Resistant Melanoma Cells
Source: Med Sci (Basel). 2019 Mar 22;7(3):51. doi: 10.3390/medsci7030051 (PMC6473314; doi:10.3390/medsci7030051)
Supplement: Supplementary file 1 [file medsci-07-00051-s001.pdf]

medical sciences

MDPI

Supplementary Materials

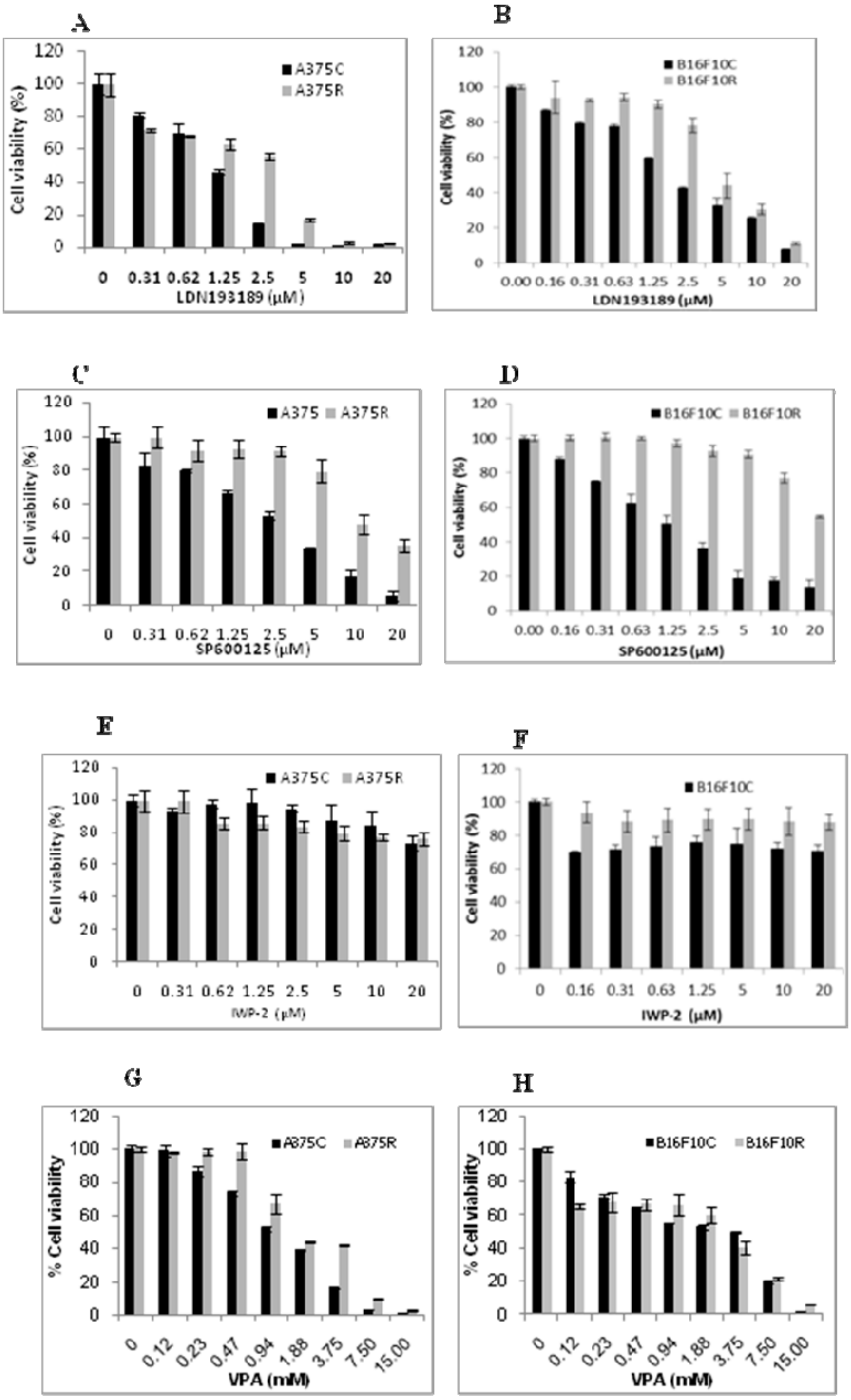

Figure 1. Cytotoxic effect of LDN193189, SP600125 IWP-2, and VPA on A375 and B16F10 cell lines. The drug concentration ranges were 0.3–20 µM for LDN193189, SP600125 and IWP2 and 0.1–15 mM for VPA. A graph was plotted with concentration of the drug (X-axis) vs. cell viability % (Y-axis) and the IC_{50} values were calculated using the formula $y = b + ax$. Data shown are means ± SEM of three replicate wells. An IC_{50} value is the concentration of the drug which results in 50% cell death. The dark bars represent naïve parental cell lines (A375C or B16F10C) and the light grey bars represent drug resistant sublines (A375R or B16F10R).

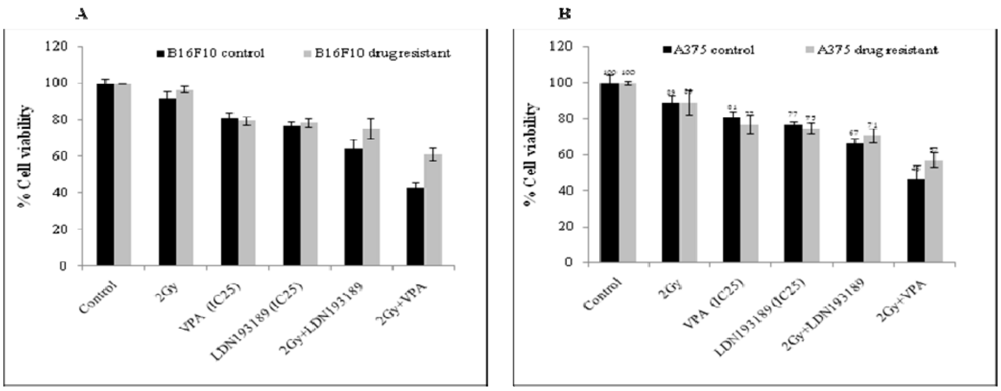

Figure S2. Radiation sensitization effects of VPA and LDN193189 on A375 and B16F10 cell lines. A375 and B16F10 cells, both parental and resistant, were pre-treated with IC_{25} dose of VPA or LDN193189. After 24 h of incubation, the flask containing cells were irradiated with a gamma radiation (GC-5000, BRIT Mumbai, India) at the CARRT, Mangalore University, at a dose rate of 8.8 Gy/min for 14 s (equivalent to 2 Gy) at room temperature. After irradiation, cells were incubated further for 24 h with drugs and MTT assay was performed. Respective sham irradiation and control groups were used for comparison and data shown are mean ± SEM of three replicate wells. The results of MTT assay: (**A**) human melanoma A375 and (**B**) mouse melanoma B16F10; control (dark bars) and resistant (light bars).
